# Mathematical models of iron metabolism: structure and functions

**DOI:** 10.18699/vjgb-25-108

**Published:** 2025-12

**Authors:** N.I. Melchenko, I.R. Akberdin

**Affiliations:** Novosibirsk State University, Novosibirsk, Russia; Novosibirsk State University, Novosibirsk, Russia Institute of Cytology and Genetics of the Siberian Branch of the Russian Academy of Sciences, Novosibirsk, Russia Research Center for Genetics and Life Sciences, Sirius University of Science and Technology, Sirius Federal Territory, Krasnodar region, Russia

**Keywords:** mathematical modeling, iron metabolism, ferritin, hepcidin, ordinary differential equations, математическое моделирование, метаболизм железа, ферритин, гепсидин, обыкновенные дифференциальные уравнения

## Abstract

Mathematical models represent a powerful theoretical tool for studying complex biological systems. They provide an opportunity to track non-obvious interactions and conduct in silico experiments to address practical problems. Iron plays a key role in oxygen transport in the mammals. However, a high concentration of this microelement can damage cellular structures through the production of reactive oxygen species and can also lead to ferroptosis (programmed cell death associated with iron-dependent lipid peroxidation). The immune system contributes greatly to the regulation of iron metabolism – hypoferritinemia (decreased ferritin concentration in the blood) during infection –which is a result of the innate immune response. In the study of iron metabolism, many aspects of regulation remain insufficiently studied and require a deeper understanding of the structural-functional organization and dynamics of all components of this complex process in both normal and pathological conditions. Consequently, mathematical modeling becomes an important tool to identify key regulatory interactions and predict the behavior of the iron metabolism regulatory system in the human body under various conditions. This article presents a review of iron metabolism models applicable to humans presented in chronological order of their development to illustrate the evolution and priorities in modeling iron metabolism. We focused on the formulation of numerical problems in the analyzed models, their structure and reproducibility, thereby highlighting their advantages and drawbacks. Advanced models can numerically simulate various experimental scenarios: blood transfusion, signaling pathway disruption, mutation in the ferroportin gene, and chronic inflammation. However, existing mathematical models of iron metabolism are difficult to scale and do not account for the functioning of other organs and systems, which severely limits their applicability. Therefore, to enhance the utility of computational models in solving practical problems related to iron metabolism in the human body, it is necessary to develop a scalable and verifiable mathematical model of iron metabolism that considers interactions with other functional human systems (e. g., the immune system) and state-of-the-art standards for representing mathematical models of biological systems

## Abstract (ru)

Математические модели представляют собой мощный теоретический инструмент для изучения сложных биологических систем. Они позволяют прослеживать неочевидные взаимодействия и проводить виртуальные эксперименты для решения практических задач. Железо играет ключевую роль в транспорте кислорода в организме млекопитающих. В то же время высокая концентрация этого микроэлемента может повреждать клеточные структуры за счет продукции активных форм кислорода, а также привести к ферроптозу (программируемая клеточная гибель в связи с железо-зависимым перекисным окислением липидов). Большой вклад в регуляцию метаболизма железа вносит иммунная система: гипоферритинемия (снижение концентрации ферритина в крови) на фоне инфекции является результатом врожденного ответа иммунной системы. В исследовании метаболизма железа многие аспекты регуляции остаются недостаточно изученными; требуется более глубокое понимание структурно-функциональной организации и динамики всех компонентов этого комплексного процесса в норме и при патологии. Важным инструментом, позволяющим выявить наиболее существенные регуляторные взаимодействия и предсказать поведение метаболической системы регуляции железа в организме человека в разных условиях, становится математическое моделирование. Данная работа представляет обзор моделей метаболизма железа, применимых к человеку, в порядке их создания, что позволяет оценить историю развития и приоритеты в моделировании метаболизма железа. Мы акцентировали внимание на постановке численных задач в анализируемых моделях, их структуре и воспроизводимости, на основе чего выделили их недостатки и преимущества. Современные модели способны численно воспроизвести множество экспериментов: гемотрансфузию, нарушение сигнального пути; мутацию в гене ферропортина; хроническое воспаление. Однако существующие математические модели метаболизма железа сложно масштабировать, и они не учитывают работу других органов и систем, в связи с чем их применение остается крайне ограниченным. Для расширения применимости компьютерных моделей в решении практических задач, связанных с метаболизмом железа в организме человека, необходимо создать масштабируемую и верифицируемую математическую модель метаболизма железа с учетом взаимодействия с другими функциональными системами человека (например, иммунной) и современных стандартов представления математических моделей биологических систем.

## Introduction

Iron plays a key role in oxygen transport in vertebrate organisms (Pantopoulos et al., 2012). In the human body, iron exists in multiple forms (Vogt et al., 2021). In blood plasma, iron is transported both in a free, transferrin-unbound form and in a transferrin-bound form, as part of hemoglobin. Iron is predominantly found in tissues either in a free form or bound to the iron storage protein ferritin. However, the majority of iron in the body is present in erythrocytes as hemoglobin.

Both iron excess and deficiency lead to adverse consequences. Iron deficiency results in iron-deficiency anemia, while iron overload causes toxic effects of free iron and triggers programmed cell death mediated by iron – ferroptosis (Xie et al., 2016). Therefore, vertebrates have a molecular genetic system orchestrating iron homeostasis. The main protein regulating iron metabolism is hepcidin. It binds to ferroportin (FPN), a protein that functions as the sole iron exporter in vertebrates. Hepcidin binding leads to ubiquitination, internalization, and degradation of FPN, thereby inhibiting iron export. Since FPN is highly expressed in duodenal enterocytes, iron-recycling macrophages, and hepatocytes, hepcidin-mediated inactivation and degradation of FPN reduce dietary iron absorption and limit the release of stored iron, thus lowering circulating iron levels (Xu et al., 2021). Hepcidin expression, in turn, is controlled by negative feedback from iron concentrations both in plasma and hepatocytes, as well as by the inflammatory response, predominantly mediated by IL-6 activity (Nemeth, Ganz, 2023).

Currently, many aspects of iron metabolism remain incompletely understood – for example, non-heme iron transport into enterocytes, allosteric regulation of hemoglobin, and hepcidin regulation (Ahmed et al., 2020; Nemeth, Ganz, 2023). Since experimental approaches cannot thoroughly uncover the complexity and hierarchical organization of the system of interacting components regulating iron metabolism in the human body, the reconstruction of a comprehensive model of iron metabolism that accounts for molecular interactions between various organs and systems will not only integrate these organizational levels of the molecular genetic iron metabolism system within a unified conceptual framework but also serve as a theoretical basis for in silico studies aimed at investigating the structural-functional organization and dynamics of interactions among system components. This, in turn, will provide a foundation for the development and evaluation of drug efficacy targeting various therapeutic sites within the iron metabolism system, considering functional interactions with the immune system.

Herein, we review existing models, assessing their advantages and disadvantages as well as their applicability in addressing fundamental and applied aspects of iron metabolism research.

## Initial models of iron metabolism in the human body

**Mathematical model of iron metabolism (Franzone et al., 1982)**

**Fig. 1.**
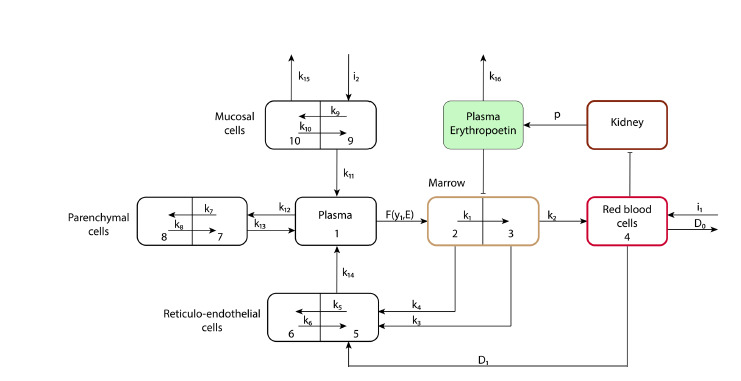
Schematic representation (adapted from Franzone et al., 1982). In the figure, the blocks represent the amount of iron in a specific organ or system, where 1 – blood plasma, 2 – maturing erythroid blood cells, 3 – mature erythroid blood cells, 4 – erythrocytes, 5 – macrophages, 6 – iron storage in macrophages, 7 – extravascular fluid, 8 – iron storage in hepatocytes, 9 – intestinal epithelial cells, 10 – iron storage in intestinal epithelial cells. The arrows indicate iron transport between organs and systems, where k1, k2, k3… k16 are the rates of iron transport between the blocks, i1 – iron influx due to blood donation, i2 – iron influx from food, D0 – iron loss due to bleeding, D1 – transfer of iron to reticuloendothelial system cells as a result of phagocytosis, F(y1, E) – function describing the transfer of iron from plasma to erythroid cells, where y1 is the amount of iron in the blood plasma, and E is the amount of erythropoietin, p – function of erythropoietin synthesis.

The model developed by P.C. Franzone and colleagues was designed to numerically estimate the concentration of iron in various compartments of the body, as well as to study the effects of different treatment methods on patients with anemia of various origins. The metabolic processes in the model are distributed across the following compartments: intestinal mucosa, blood plasma, liver, reticuloendothelial cells, bone marrow, and erythrocytes. The model describes the intake of iron from food, its transport into plasma, storage in the liver, and participation in erythropoiesis. It takes into account the impact of erythropoietin on the proliferation and maturation of erythroid cells. The model also allows for the consideration of iron replenishment through donor blood and iron loss due to bleeding. To account for the process of iron return from erythrocytes to blood plasma, the model includes a component describing the destruction of erythrocytes by reticuloendothelial cells. Additionally, the model considers ineffective hematopoiesis, whereby some erythroid cells fail to complete differentiation (Fig. 1).

The model simulations were conducted on conditions such as blood donation in a healthy patient, blood transfusion after splenectomy in a patient with hemolytic anemia, as well as treatment of hypoplastic anemia using transfusions and androgens than two months, which corresponded to the literature data at the time of publication (Wadsworth, 1955; Liedén et al., 1975) and also aligns with data from recent studies (Kiss et al., 2015; Ziegler et al., 2015).

The model was also used to numerically investigate blood transfusion after splenectomy (removal of the spleen). The resulting model more accurately describes iron dynamics for patients after splenectomy. However, data from only one patient were used to validate this condition

The proposed model was also used to study the effect of treating hypoplastic anemia with transfusions and androgens. However, these results have lost their relevance since such therapy is no longer used today (Killick et al., 2016). The authors of the developed model note that the system’s equations can exhibit stiff behavior due to the large differences between the numerical values of transport rates when modeling anemic conditions. Considering the stiffness of the system, to achieve a compromise between accuracy and computational resources, the authors used the implicit trapezoidal method for the numerical solution of the system (Tavernini, 1973).

Given that Franzone and co-authors’ model is one of the first models describing iron metabolism, it is significantly inferior to modern models. This model lacks descriptions of key participants in iron metabolism: hepcidin, ferritin, transferrin, and proteins regulating the expression of genes involved in iron metabolism (Iron Regulatory Proteins, IRP). The iron storage process is greatly simplified and represented by a linear coefficient. Despite this, the authors managed to simulate complex conditions such as blood transfusion after splenectomy in a patient with hemolytic anemia and treatment of hypoplastic anemia using transfusions and androgens. However, considering that data from only one patient was used to validate the numerical calculations of the model for each of these conditions, it is difficult to assess how applicable the numerical modeling results are to population data and how parameters might change when reproducing data on other patients.

**Computational model of iron metabolism in the liver (Mitchell, Mendes, 2013**

**Fig. 2.**
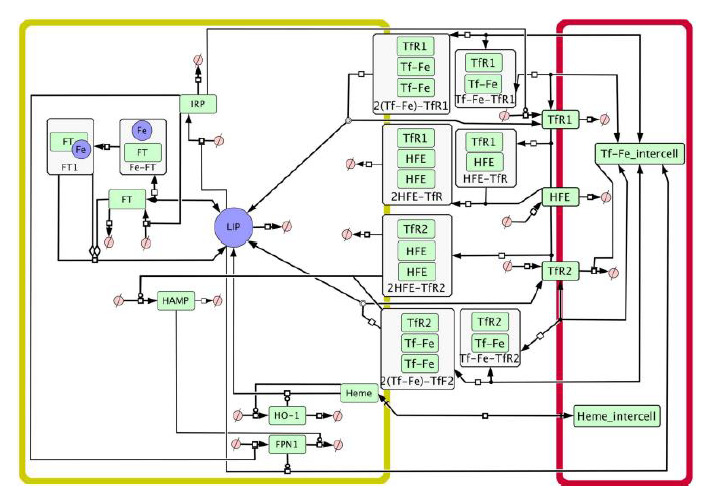
Graphical representation of the model in the SBGN standard (Le Novère et al., 2009). Arrows designate substance transport. Yellow compartment – hepatocyte, red compartment – plasma, LIP – labile iron pool, FT – ferritin, Fe – iron, HAMP – hepcidin, Heme – heme, HO-1 – heme oxygenase 1, IRP – iron regulatory proteins, FPN1 – ferroportin, TfR1 – transferrin receptor 1, TfR2 – transferrin receptor 2, Tf-Fe_intercell – plasma transferrin-bound iron (Mitchell, Mendes, 2013).

The mathematical model proposed by S. Mitchell and P. Mendes in 2013 allows the numerical evaluation of processes related to iron transport into hepatocytes. The model enables quantitative prediction of the concentration of proteins synthesized in the liver that regulate iron metabolism. The model consists of 21 ordinary differential equations and includes two compartments: hepatocyte and plasma (Fig. 2).

Using the model built, the authors numerically analyzed the following physiological conditions: hereditary hemochromatosis types 1 and 3. To reproduce the state of type 1 hemochromatosis, a virtual knockdown of the human iron homeostasis regulator protein (HFE) was performed by reducing the synthesis constant 100-fold. The model could not quantitatively reproduce the result that mice with this pathology have liver iron levels three times higher than normal. This was due to the fixed concentration of intercellular transferrin-bound iron in the model, unlike that in mice, which show increased transferrin saturation as a result of increased intestinal iron absorption. Despite fixed extracellular conditions, the model predicts intracellular iron overload in hepatocytes. The hemochromatosis model also reproduced the dynamics observed in experiments with changes in dietary iron content. Increased dietary iron doubled ferroportin expression in the liver in both healthy mice and those with hemochromatosis. To reproduce the state of type 3 hemochromatosis, a virtual knockdown of TfR2 was performed, also by reducing the synthesis constant 100-fold. Numerical analysis revealed an increase in hepcidin concentration and a decrease in ferroportin concentration, which was consistent with experimental data (Chua et al., 2010)

The model describes the iron transport into hepatocytes well, considering iron storage, export, and utilization for heme synthesis. We also comprehensively reproduced the authors’ results both in the COPASI software (Hoops et al., 2006) and in the BioUML platform (Kolpakov et al., 2022). However, the model has some limitations: (1) the model lacks an important regulatory link in iron metabolism, namely the effect of hepcidin on iron absorption from the intestine; (2) fixed concentrations of heme and intercellular transferrin-bound iron are used; (3) due to limited availability of quantitative clinical data on human iron metabolism, various other data sources were integrated for parameterization, such as in vitro experiments and animal models; (4) the parameters reported in the study do not correspond to the model parameters in the supplementary material.

**Modeling of the system iron regulation in various pathologies considering hepcidin-independent mechanisms (Enculescu et al., 2017)**

The model by M. Enculescu and colleagues (2017) describes iron metabolism throughout the human body, taking into account intra- and extracellular regulatory mechanisms of iron metabolism. The authors focused primarily on the system regulation of iron metabolism via the hepcidin-ferroportin regulatory axis. The model describes iron content in seven compartments: serum, liver, spleen, bone marrow, erythrocytes, duodenum, and “other organs,” representing iron distribution in the mouse body. Iron absorption and loss in the duodenum, as well as iron loss in the “other organs” compartment, are considered. The model explains inhibition of ferroportin transcription during inflammation and regulation of its translation by intracellular iron, as well as hepcidinmediated post-translational destabilization of ferroportin. Iron export from peripheral organs is controlled by the iron exporter ferroportin (Fpn), which is predominantly localized on the plasma membrane of three cell types: duodenal enterocytes, macrophages, and hepatocytes. Fpn expression is described separately for each organ and regulated by three mechanisms: (1) inflammatory signals decrease Fpn mRNA transcription; (2) intracellular iron enhances Fpn mRNA translation; (3) Fpn protein turnover is increased by the soluble polypeptide hepcidin.

**Fig. 3.**
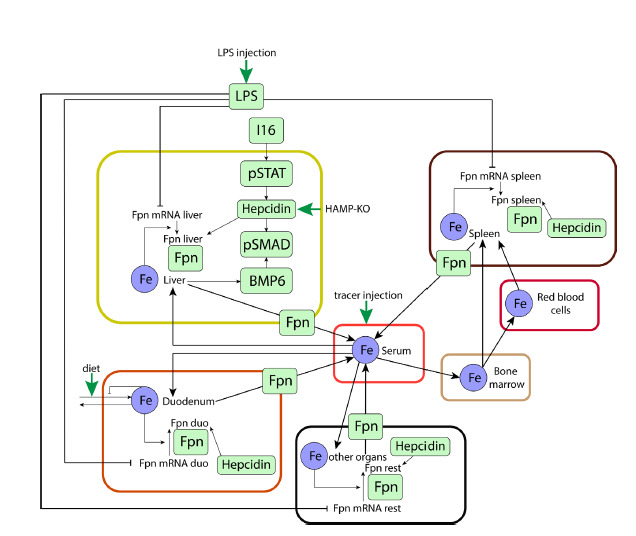
Graphical representation of the model (Enculescu et al., 2017) in the SBGN standard (Le Novère et al., 2009). LPS – lipopolysaccharides, Fpn – ferroportin, BMP6 – bone morphogenetic protein (regulatory protein), pSMAD, pSTAT – transcription factors. Black arrows indicate substance transport, green arrows designate substance input from outside the organism.

Hepcidin expression is activated by the iron-sensitive BMP6/SMAD pathway and an inflammatory signaling cascade involving cytokine production (primarily IL-6) and subsequent phosphorylation of the transcription factor STAT3 in hepatocytes (Fig. 3).

The authors’ own data and previously published data were used for the model calibration. A total of 344 experimental measurements were obtained. The following assumptions were made for model parameterization: in some cases, homologous reactions in different compartments proceed with identical kinetic rate constants. Additionally, kinetic parameters of the hepcidin gene promoter model were fixed at values previously determined by the authors in the HuH7 cell culture system.

The following conditions were numerically investigated using the constructed model: administration of lipopolysaccharides (LPS) under iron overload; disruption of the BMP6 signaling pathway; mutation in the ferroportin gene leading to loss of ferroportin’s ability to bind hepcidin; chronic inflammation.

The authors also used data from their own experiment to validate the model in the numerical analysis of LPS administration under iron overload. According to the experiment, male C57BL/6 mice were fed an iron-rich diet containing 100 times more iron than a normal diet for four weeks, followed by a single dose of LPS at 1 μg/kg. The experimental data corresponded to the model’s predictions for most variables: iron in serum, liver, and duodenum; hepcidin content in the liver; BMP6 mRNA concentration; levels of pSTAT and pSMAD in the liver; mRNA and protein content of ferroportin in the liver. Deviations of the model approximation from experimental data were observed in the following indicators: iron content in the spleen and erythrocytes, ferroportin concentration in the spleen.This study also provides a numerical analysis of the dynamic behavior of the iron regulation system when hepcidin feedback is blocked. Two situations were reproduced for this: (1) disruption of the BMP6 signaling pathway; (2) mutation in the ferroportin gene leading to the loss of ferroportin’s ability to bind hepcidin.

To reproduce the first condition, SMAD expression was set to zero, whereas to reproduce the second condition, the parameter values describing hepcidin’s effect on ferroportin degradation were also set to zero. Numerical simulations of the model in both cases showed an increase in iron concentration in the serum and liver and a decrease in iron concentration in the spleen, which was confirmed by experimental data. Moreover, as in the experiments, ferroportin resistance to hepcidin led to increased hepcidin expression, whereas the loss of SMAD signal transduction caused a significant decrease in hepcidin expression

Then the authors hypothesized that hepcidin affects ferroportin in a tissue-specific manner. To model this situation, the authors sequentially set to zero the parameter values describing hepcidin’s effect on ferroportin degradation in different tissues. The results of the numerical analysis demonstrated that only the elimination of hepcidin-mediated regulation of ferroportin in the duodenum has a system effect, leading to an increase in iron concentration in other organs. Meanwhile, modeling ferroportin resistant to hepcidin in the liver or spleen leads only to a local effect with a decrease in iron stores in the corresponding organ and minimal changes in other organs. Mouse models with tissue-specific resistance to hepcidin have not yet been described. However, tissue-specific deletion of FPN in intestinal cells has been studied in mice. This study showed that deletion of FPN in intestinal cells leads to severe iron deficiency in blood, liver, and spleen.

The research team of the proposed model also applied it to conduct an in silico experiment studying chronic inflammation. Equations describing the kinetics of LPS and their effect on hepcidin were added to model the scenario. Numerical analysis of the model describing persistent inflammation showed an 85 % decrease in serum iron concentration; iron concentration in erythrocytes decreased over a longer period, stabilizing after two months at a value equal to 10 % of the normal level.

This investigation considers two mechanisms of ferroportin regulation: at the transcript level and regulation by hepcidin. To assess the contribution of each regulatory path, the authors modeled LPS responses when either the transcriptional or post-translational effect of LPS on ferroportin protein levels was eliminated. Numerical analysis indicated that the absence of hepcidin influence during inflammation resulted in a normal decrease in serum iron level (75 % of the original model version). In contrast, removal of transcriptional control of ferroportin during inflammation reduced hypoferriemia to 50 %. The authors concluded that removal of transcriptional control of ferroportin causes greater deviations in serum iron values from normal than removal of hepcidin control. This concludes that hypoferriemia arises as a result of a combination of hepcidin-dependent and independent mechanisms.

Among the limitations of the proposed model, the authors note varying degrees of parameter accuracy and the absence of description of iron binding to ferritin and its storage.

## Erythropoiesis and iron metabolism model in humans (Schirm, Scholz, 2020)

**Fig. 4.**
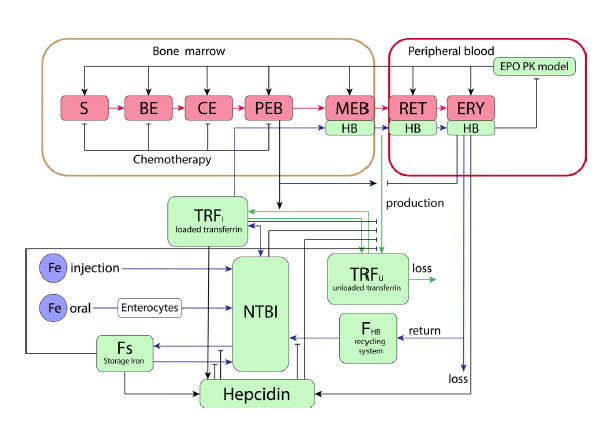
Graphical representation of the model (Schirm, Scholz, 2020) in the SBGN standard (Le Novère et al., 2009). S – stem cells, BE – burst-forming unit, CE – colony-forming unit, PEB – proliferating erythroblasts, MEB – maturing erythroblasts, RET – reticulocytes, ERY – erythrocytes, HB – hemoglobin, NTBI – non-transferrin-bound iron. Blue arrows indicate iron flow, green arrows represent transferrin flow, red arrows show the differentiation progression of erythroid lineage cells, and black arrows denote regulatory influences.

A group of authors from the University of Leipzig developed a mathematical model (Schirm, Scholz, 2020) aimed at predicting the effects of treatments involving unproven therapeutic options, such as cytotoxic chemotherapy supported by iron and erythropoietin (EPO). The model is an extension of the authors’ previous study on erythropoiesis modeling (Schirm et al., 2013), which was expanded by adding an extra module for iron metabolism. The original erythropoiesis module describes the dynamics of erythropoietic cell development, reflecting all the main stages of differentiation: stem cells, burst-forming units, colony-forming units, proliferating erythroblasts, maturing erythroblasts, and reticulocytes. This module also accounts for the effects of chemotherapy on erythropoiesis. The module describing iron metabolism includes the following compartments: hepcidin, non-transferrin-bound iron (NTBI) in plasma, the hemoglobin catabolic system, iron stores, transferrin bound to iron, and free transferrin (Fig. 4).

Within the framework of computational modeling, some simplifications of the complex physiological system were employed to reduce the number of unknown model parameters or due to the lack of quantitative data for humans. The model does not consider separate pools of Fe2+ and Fe3+ concentrations due to the absence of data, nor does it specify concentrations of transferrin saturated with one or two iron ions.The following conditions were studied via the numerical analysis of the proposed model: (1) oral iron administration in healthy individuals; (2) intravenous injection of EPO with oral iron administration in healthy individuals; (3) iron deficiency; (4) intravenous iron administration in healthy individuals; (5) bleeding/phlebotomy; (6) chronic inflammation; (7) hemochromatosis.

To validated the model’s numerical calculations, the authors harnessed the data from several clinical studies with different treatment modes (Rutherford et al., 1994; Souillard et al., 1996; Kiss et al., 2015). The authors numerically investigate the experimental scenario of Souillard and colleagues (1996), in which healthy athletes received 200 IU/kg of EPO on days 0, 2, 4, 7, and 10 without iron supplementation. The obtained in silico results for the quantity or concentration of reticulocytes, hemoglobin, erythrocytes, hematocrit, and ferritin generally differ from the clinical study data by no more than one standard deviation.To validate the numerical results describing EPO administration with iron supplements, the authors used the data from by Rutherford and coauthors’ study (1994). In this clinical trial, patients received EPO at a dose of 1,200 IU/kg per week with different dosing regimens and iron at a dose of 300 mg orally daily for 10 days. The modeling results for hematocrit, reticulocyte, ferritin concentrations, and transferrin saturation reflect the dynamics of these parameters in the clinical study very well. However, the numerical results for hemoglobin are underestimated.

S. Schirm and M. Scholz also conducted a numerical experiment on the donation of 500 mL of blood, both with and without iron supplementation. To validate the numerical results, the authors employed the clinical study by Kiss et al. (2015), which provided quantitative measurements of ferritin and hemoglobin dynamics. The numerical results for ferritin concentration calculated by the model differ from the clinical data by no more than one standard deviation in both scenarios, while the numerical results for hemoglobin dynamics in the iron supplementation scenario differ from the clinical data by more than one standard deviation over a large interval.

This study also included a virtual experiment aimed at a theoretical prediction for unused therapy. The Scholz group modeled the effect of CHOP-14 therapy supported by iron supplements and EPO on erythropoiesis and iron metabolism. CHOP-14 is a commonly accepted therapy for treating aggressive non-Hodgkin lymphomas, including drugs such as doxorubicin, cyclophosphamide, vincristine, and prednisolone. Currently, the therapy has been extended to R-CHOP, which also includes rituximab (Phan et al., 2010). This therapy is hematotoxic, so the authors considered the possibility of supplementing it with iron and EPO. To validate the numerical results in the in silico experiment of chemotherapy without iron and EPO supplementation, the data from a German research group on high-grade non-Hodgkin lymphoma (Pfreundschuh et al., 2004) were used. According to the numerical results of the in silico experiment, adding iron supplements together with EPO in patients undergoing CHOP-14 therapy slowed the decline in hemoglobin concentration. When iron supplements and EPO are administered on days 3, 7, and 21, the hemoglobin concentration on day 80 is approximately 11.2 g/dL, whereas without supportive therapy it is about 10.7 g/dL. With weekly administration of iron supplements together with EPO starting from day 45, hemoglobin concentration recovers to 12.5 g/dL by day 80, while without supportive therapy hemoglobin concentration falls to 10.7 g/dL. It is important to note that EPO plays a significant role in hemoglobin recovery, as numerical results for supportive therapy with iron supplements alone practically did not differ from those without it.

The authors adhered to a modular approach and built the model upon their previous study by adding new components. A major advantage of this study is the validation using a large amount of data from various studies. The model demonstrated good agreement with clinical trials, as in most cases the differences between the model’s numerical data and clinical results did not exceed one standard deviation. One drawback is the lower hemoglobin level predicted by the model compared to experimental measurements.

## Model of iron sequestration by ferritin (Masison, Mendes, 2023)

P. Mendes and J. Masison developed a model describing the binding of iron ions by the protein ferritin. Ferritin consists of 24 subunits and is capable of binding about 4,300 iron atoms per ferritin molecule. Ferritin is an important participant in iron metabolism, so iron exchange models must include it. Such a model enables integrating the interaction of ferritin with iron ions into more complex models.

The model considered: (1) how iron bound to ferritin affects the dynamics of iron sequestration; (2) how the iron sequestration model with rate constants obtained experimentally in vitro can numerically reproduce experimental results obtained in cell lines; (3) the influence of ferritin subunit composition on the rate of iron sequestration; (4) the dependence of iron release dynamics from ferritin on the concentration of free iron and ferritin in the cell.

**Fig. 5.**
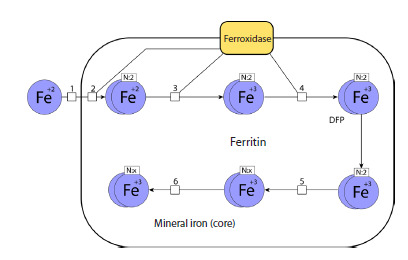
Adapted graphical representation of the iron sequestration model (Masison, Mendes, 2023) in the SBGN standard (Le Novère et al., 2009). The following reactions are shown: 1 – transport of Fe2+ into ferritin, 2 – binding of Fe2+ with ferroxidase, formation of DFP, 3, 4 – oxidation of Fe2+, 5 – nucleation, 6 – mineralization.

The model accounted for four chemical species: LIP – labile iron pool, soluble or readily soluble divalent iron in the cytoplasm; DFP – peroxo complex containing two iron atoms; core – iron incorporated into the mineralized ferrihydrite core; FT – 24 subunits of ferritin. The model included four reactions, three of which describe the process of iron sequestration by ferritin: oxidation converts two LIP into one DFP; nucleation converts two DFP into a new crystal core; mineralization adds one DFP to an existing core; and one reaction describes degradation of the intermediate product: reduction converts one DFP back into two LIP. The sequestration process is shown schematically in Fig. 5. The authors simplify and combine several of its components to construct a system of differential equations that reflects this biochemical process with sufficient accuracy. At the same time, they avoid excessive details and do not overload the model with variables

The first reaction describes the oxidation of LIP to DFP and is represented by a Hill function

**Reaction. 1.**
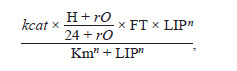
Reaction 1

kcat – catalytic turnover number, Km – Michaelis constant, n – Hill coefficient. The ferritin molecule consists of 24 subunits of two different types, H and L, and only the H subunits contain the active ferroxidase center. Therefore, molecules with different subunit compositions have different oxidation rates. To account for this, two additional parameters were used: H – the number of the H subunits (a value from 0 to 24); rO – a scaling factor representing the oxidation efficiency of the L homopolymer.

The parameter rO was included by the model authors because, despite the L subunits lacking a known ferroxidase, the L homopolymers still catalyze the formation of ferric iron (Fe3+) within ferritin according to experimental data, although at a rate reduced by more than a quarter (Carmona et al., 2014). Since data on how oxidation occurs in the absence of the H subunit and the corresponding value of rO are limited, the value of rO was empirically set to two.

The second reaction, degradation of DFP, follows the law of mass action:

**Reaction. 2.**
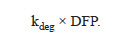
Reaction 2

The third reaction is nucleation:

**Reaction. 3.**
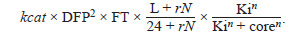
Reaction 3

It describes the process of forming a new crystal from two DFP molecules inside the ferritin molecule. New nuclei can also form within a ferritin molecule that already contains an existing core. The equation was empirically derived based on the law of mass action. The coefficients L and rN reflect how the ferritin subunit composition influences nucleation; due to limited information on this process, the coefficient rN was chosen to contribute significantly less to the nucleation rate range than the coefficient rO does to the oxidation rate range. The last factor included an inhibition constant and a Hill coefficient, allowing for the decrease in the probability of new crystal formation as the size of the existing core increases.

The fourth reaction is mineralization:

**Reaction. 4.**
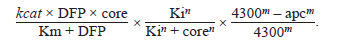
Reaction 4

The published data (Harrison et al., 1974) demonstrated that the rate of this reaction reaches a maximum at 1,500– 2,000 iron atoms per core and decreases with further core growth. The second factor is needed to account for this process, while the third factor drives the rate to zero as the number of iron atoms per core (apc) approaches the maximum allowable value of 4,300.

To validate the model simulations, experimental data from different laboratories under various conditions were used. The model exhibited some differences compared to experimental data within the first 20 seconds: a stronger cooperative effect in the DFP mineralization rate and a faster attainment of steady-state concentration. Since the model’s target context is cellular models, where the relevant time scale is minutes or longer, such differences from experimental data are not considered significant.

The authors conducted a virtual experiment investigating the influence of iron atoms in the core on the mineralization rate. The simulation revealed that the mineralization rate over time depends on the initial number of iron atoms per core (apc). Typically, the curves showing mineralization rate fall into three groups based on the initial apc. In the first group (<1,000 apc), the mineralization rate starts low, then increases as iron accumulates inside ferritin, and later decreases as the iron concentration in the solution drops. In the second group (1,000–3,000 apc), the mineralization rate starts high but rapidly declines due to decreasing iron concentration in the solution. Eventually, in the third group (>3,000 apc), the mineralization rate decreases throughout the simulation, as iron accumulation in the ferritin core slows down further mineralization.

Then the authors investigated the model behavior at ferritin and iron concentrations corresponding to those found in mammalian cells. The research team led by Mendes incorporated this model as a modular component into their previously developed model of iron metabolism in hepatocytes. The authors reported that the system’s qualitative behavior remains similar to the original model before extension. However, the expanded model provided a deeper understanding and better assessment of iron storage mechanisms. Due to the increased detail of the new model, it becomes clear that the peak in ferritin-bound iron is driven by an increase in the concentration of DFP rather than the mineralized core – an important distinction since DFP is more readily released back into the cytoplasm. The numerical results of the models differed both over the time course and at equilibrium. The greatest differences appear after 1,000 seconds of simulation. In the original model, ferritin-bound iron content gradually increased, whereas in the new model, its concentration decreased. The authors of the original study hypothesized that this discrepancy may be related to new iron storage kinetics, which promotes a reduction in available iron through ferritin buffering, whereas in the original model, other mechanisms primarily influenced the kinetics of available iron.

## Conclusion

The analysis of the presented mathematical models of iron metabolism reveals a tendency toward a progressive increase in their structural complexity over time (Supplementary Table S1)1. With the advancement of research, both the number of equations and the number of parameters in the models grow, indicating a pursuit of a more accurate and detailed description of biological processes. More recent models provide the simulation of a broader range of physiological and pathological states, expanding the possibilities for conducting in silico experiments. An exception is the latest model of iron sequestration by ferritin (Masison, Mendes, 2023), which is implemented according to a modular principle and was developed with the aim of integration into more complex systems. This approach ensures the flexibility and scalability of the model, which is important for further development and incorporation into multifactorial models of iron metabolism.


Supplementary Materials are available in the online version of the paper: https://vavilovj-icg.ru/download/pict-2025-29/appx38.pdf


To deeper understand the iron metabolism, it is necessary to consider its interaction with the immune system, as it plays a key role in regulating iron homeostasis (Vogt et al., 2021). At the same time, the reduction of iron availability to pathogens and the production of reactive oxygen species can significantly affect the dynamics of infectious diseases (Weinberg, 2009). Inclusion of these factors in mathematical models will enable virtual experiments analyzing the impact of various infections on iron metabolism and assessing the long-term consequences of such interactions. This knowledge may be critically important for developing new approaches to treat diseases associated with iron metabolism disorders, as well as for understanding the pathogenesis of conditions such as anemia under chronic diseases, hemochromatosis, or post-viral syndromes, such as post-COVID syndrome.

Thus, integrating data on the interactions between the immune system and iron metabolism will not only deepen our understanding of these processes but may also pave the way for new opportunities for clinical research and therapeutic strategies. In this regard, the construction of a detailed model of iron metabolism that takes into account its interactions with the immune system represents a timely task, the solution of which will enable better understanding of the interplay between these two complex systems and allow the identification of key links in the pathology of iron metabolism in various diseases.

## Conflict of interest

The authors declare no conflict of interest.
